# Physicochemical properties, biological chemistry and mechanisms of action of caries-arresting diammine-silver(I) fluoride and silver(I)-fluoride solutions for clinical use: a critical review †

**DOI:** 10.3389/froh.2024.1412751

**Published:** 2024-07-23

**Authors:** Kayleigh Hunwin, Georgina Page, Mark Edgar, Adolfo Botana, Rachel Armitage, Mohammed Bhogadia, Unmesh Desai, Steven Duffin, Marcus Duffin, Wyman Chan, Martin Grootveld

**Affiliations:** ^1^ Leicester School of Pharmacy, De Montfort University, The Gateway, Leicester, United Kingdom; ^2^ JEOL (U.K.) Ltd., Welwyn Garden City, United Kingdom; ^3^ Shoreview Dental LLC, Keizer, OR, United States; ^4^ NoDK LLC, Wilsonville, OR, United States; ^5^ Oral Health Outreach LLC, Wilsonville, OR, United States; ^6^ SmileStudio (U.K.) Ltd., London, United Kingdom

**Keywords:** dental caries, human saliva, diammine-silver(I) fluoride (SDF), silver(I)-fluoride (SF), silver(I)-chloride (AgCl), chromophoric Ag/AgCl-based nanoparticles (CSNPs), IV-SCAN, mechanisms of action (MoAs)

## Abstract

This paper serves as a Part II follow-up of our research investigations performed on the molecular structures of silver(I)-fluoride (SF) and diammine-silver(I) fluoride (SDF) complexes in solution-based commercial products for clinical application, their precise chemical compositions, and their nature in aqueous solution, the latter including rapid fluoride-exchange processes at the silver(I) ion centre monitored by ^19^F NMR analysis (Part I). Part I of this series also explores the mechanisms of action (MoA) of these complexes, and is therefore largely focused on their chemical reactions with constituents of human saliva, which has access to their sites of application. Such reactions were found to slowly promote the generation of potentially physiologically-active Ag/AgCl nanoparticles from primarily-generated discoloured silver(I) chloride (AgCl) precipitates, a process involving salivary electron-donors such as thiocyanate and L-cysteine. Since this research has shed new light on potential MoAs for these products, in this accompanying report (Part II), we have performed a critical review of scientific literature in order to rationalize our results in relation to current views on these mechanisms for SF and SDF products employed for the successful clinical arrest of dental caries. Following an Introduction to the subject matter ( Section 1), this paper comprises a generalized overview of silver coordination chemistry ( Section 2), which is followed by a section focused on the aqueous solution status and equilibria involved in SF chemistry ( Section 3), the latter including results acquired from an original simulation of the electronic absorption spectra of coloured SF complexes in aqueous solution (Section 3.1). Section 4 then investigates detailed rationales for the biologically-relevant ligand-exchange and redox chemistries, disposition and fates of SF, SDF and silver(I)-nitrate when employed for the treatment of dental caries, with emphasis placed on their therapeutic MoAs. This Section is supported by the provision of valuable information centralized on (1) relevant biomolecular chemistry involved in solution- and solid-state matrices ( Section 4.1); (2) SF and perhaps silver(I)-nitrate as more cost-effective alternatives to SDF therapies ( Section 4.2); and (3) the potential therapeutic benefits and effects offered by silver-based nanoparticles and their associated MoAs ( Section 4.3). Recommendations for future investigations in this area are proposed.

## 1 Introduction

For some time now, dental clinicians have employed diammine-silver(I) fluoride (SDF) and silver(I)-fluoride (SF) as anti-caries agents on a global basis, albeit predominantly the former (1). Indeed, these products are valuable for the arrest of cavitated dentine carious lesions and also the circumvention of new caries developments (2). In addition to their caries-arresting and management properties ((including early childhood, root, pit and fissure, and secondary caries conditions), they may also be utilised to remineralise hypomineralised teeth (3), treat infected root canals (4), and desensitise hypersensitive teeth (5). Prior to continuing, it is important to note that the frequently-employed name “silver diamine fluoride” is a misnomer; the correct chemical nomenclature term is “diammine-silver(I) fluoride” since it features a central silver(I) ion which is virtually-linearly coordinated by two ammonia and *not* amine ligands. However, since it is commonly abbreviated as “SDF” in the scientific literature, this abbreviations is used throughout this paper.

Since SDF represents a safe, effective, efficient and equitable caries control agent, it has been described as a silver-fluoride “bullet” for the control of dental caries (6). Indeed, in 2021, the World Health Organization (WHO) included SDF as “one of the most efficacious, safe and cost-effective medicines” for addressing the most important requirements for a dental health system applicable to both adults and children (7). The WHO considers that SDF is a medicine to which everyone should always have access to, and that all governments should ensure that it is available and affordable to their populations. Despite rapidly accumulating evidence for the success of SDF treatments, it has now become a highly popular and sought-after therapy, or at least it is in the more affluent Western world. However, SDF is not yet available for clinical use in certain countries. Moreover, some countries have no documented guidelines available for SDF use.

One US survey revealed a universal approval of SDF use for the arrest of caries lesions by paediatric dentistry residency programmes in 2020, although only 26% of them confirmed use of it in 2015 (8). Since 2016, the clinical use of SDF and its potential mechanisms of action (MoAs) has also been a markedly expanding research sphere, with global research interest increasing rapidly from 2016 to 2021 (9). Scientific research investigations performed on SDF have also escalated, and results acquired therefrom have been fed into clinical practice in order to enhance dental healthcare quality.

Unfortunately, there are many fewer reports available on the dental therapeutic applications of 1:1 silver(I)-fluoride (SF) rather than SDF, for example Green in 1989 (10), Gotjamanos in 1996 (11), and Turton et al. (12) in 2021. Notably, there appears to be only very limited research outputs and publications on this agent since the 2000's (13). Although there are quite a few publications available which refer to “silver fluoride” in their titles, in fact they are actually covering SDF and its actions in their subject matter.

The objectives of this communication are largely to expound upon the results acquired from our previous experimental laboratory report, Part I of this two-part series (14), based on studies of (1) the chemical constitution and aqueous solution status of SDP and SF products (mainly using ^19^F NMR analysis), and (2) their interactions with intact whole mouth salivary supernatant (WMSS) samples, a complex process primarily forming off-coloured silver(I)-chloride (AgCl) deposits which develop into chromophoric Ag/AgCl nanoparticles via a mechanism which we describe as a salivary-catalysed autoconstruction of Ag/AgCl-based nanoparticles (IV-SCAN) process; these investigations employed scanning electron microscopy (SEM), attenuated total reflection Fourier-transform infra-red (FTIR-ATR) and ^19^F nuclear magnetic resonance (NMR) analysis techniques. This nanoparticulate material is abbreviated as CSNPs (chrompophoric silver nanoparticles) in Ref (14).

These critical studies reported were performed in order to review the molecular nature and status of topically-applied clinical formulations of both SDF and SF products, and in this context such evaluations are considered to be of the utmost importance, since clinicians using these products should understand what actually is being administered to patients in terms of their molecular structural nature, any structural heterogeneities, and the presence of any deviations from the contents of agents specified by the manufacturers of such formulations, along with the presence of any impurities or contaminants therein. Since human saliva has ready access to the clinical application site of these products, also important was our investigation of their interactions with this biofluid, since these phenomena are considered to contribute towards the MoAs of these silver(I) complexes, most notably the chemical nature of the Ag/AgCl-based nanoparticulate species generated therefrom, together with any key roles that they might play regarding suppressions of the development and progression of dental caries lesions.

Notwithstanding, this paper commences with two Sections encompassing firstly an overview of silver coordination chemistry (Section 2), and secondly an outline of the aqueous solution status and possible chemical species equilibria of SDF, SF and further silver(I) complexes (Section 3) in view of their somewhat limited consideration in most scientific publications focused on the clinical benefits and potential MoAs of these novel metallo-therapies.

## 2 Overview of silver coordination chemistry

Silver, most usually as its silver(I) cation, is generally viewed to be non-toxic towards humans and other mammals, although it should be noted that certain aspects of this consideration still remain under critical scientific debate. Indeed, the clinical introduction and applications of silver nanoparticles as microbicidal agents are now frequently used as therapeutic strategies for a vast array of conditions. Nevertheless, the low level of Ag(I) ion toxicity represents one of its major benefits, especially with regard to other known metal ion therapies such as gold(I)-thiolate, and platinum(II)-ammine or -carboxylato ligand complexes, the former metal ion originally employed for the treatment of rheumatoid arthritis, the latter for many forms of cancer. A further advantage includes its power to challenge and effectively subdue drug resistance problems; indeed, it can readily combat antibiotic-resistant bacteria, fungi and parasites. However, the efficacy-dependent bioavailability of Ag(I) could present a problem, largely because without any Ag(I) cation stabilization (for example, from the addition of two molar equivalents of ammonia (NH_3_) as ligands to form the stable, near-linear [Ag(NH_3_)_2_]^+^ complex), these ions are readily deposited as silver chloride (AgCl) precipitates *in vivo* in view of the wide availability of chloride ion in biosystems (millimolar or near-millimolar levels in human saliva, for example). This [Ag(NH_3_)_2_]^+^ complex, together with a fluoride counter-ion, is currently clinically applied topically as a very powerful agent for the arrest of dental caries lesions (6, 7). Notwithstanding, Ag(I) ions readily interact with a range of biomacromolecules, particularly with protein cysteine residues. to form stable Ag(I) complexes, polymers or even chelates, and this form of chemical reaction may also be considered as one of the major basic MoAs of Ag(I) ions. One potential solution to this problem, however, is the envelopment of Ag(I) by biodegradable polymers.

Of all the known silver coordination and organometallic compounds documented, the silver atom may occur in oxidation states of Ag(I) and Ag(II), the former being by far the most frequent occurrence, with a range of metal ion-ligand geometries ranging from coordination numbers of anything from two to seven, with two (near-linear) and four (mainly tetrahedral) being the most common. The nuclearities of their complexes varies form mono- to polynuclear, with a variety of donor ligands and chelators. However, Ag(III)-ligand complexes are much rarer, and as expected, these have a square planar coordination geometry (both distortion and polymerization isomerisms have been reported for these complexes). In 1995, Holloway et al. (15) performed a classification of silver compounds, and analysed an extensive cornucopia of both crystallographic and structural data. In particular, these researchers explored correlations between bond lengths and angles, ligating atom radii and oxidation state.

Silver and gold metals have similar ionization potentials and electronic structures; however, their respective chemistries differ markedly. These differences largely arise from relativistic effects. Indeed, a stabilization resulting from a 6s orbital contraction in gold minimizes the energy gap between itself and its 5d orbitals, which also sustains an expansion. However, both effects are far more striking for gold, so that the energetic differences between its valence 5d and 6s shells are significantly lower than those of silver, which involve 4d and 5s orbitals (16, 17). Hence, gold(I) is frequently classified as a “soft”, class b type metal ion which has a high affinity for “soft” donor ligands such as those involving biological sulphur donors such as that in the side-chain of L-cysteine, and that in the central cysteine residue of the tripeptide glutathione; the great majority of gold(I) ions biodistributed within the human body are bound to such sulphur donor ligands, and when administered as gold(I) drugs for the treatment of rheumatoid arthritis, they are mainly in the form of gold(I)-thiolate complexes, such as in the disodium salt of aurothiomalate (18).

However, complexes of silver(I) ions with “harder” nitrogen donor ligands are much more abundant than they are with gold(I), the simplest being near-linear two-coordinate complexes of the type [Ag(I)L_2_], which are known to occur for a range of N-donor ligands, including ammonia (as in [Ag(I)(NH_3_)_2_]^+^), pyridine, methylamine and melamine [aromatic nitrogen donor ligands can form silver(I) aggregates with differential structural motifs (19, 20)]. Also common is complexation of silver(I) ions by phosphorus and sulphur donor atoms, most notably by those of phosphine and thiolate ligands respectively. Similarly, complexes of silver(I) with oxygen (O)-donor ligands such as carboxylic acid anions are well known (21, 22).

Although silver(I) ions are tetrahedrally-coordinated in “pure” solvent systems, ligands such as ammonia form near-linear, two-coordinate complexes, and this is explicable by the propensity of this cation towards *sd*-hybridization. To investigate this discrepancy further, Fox et al. (23) studied the reaction of ammoniated silver(I) cations [Ag^I^(NH_3_)_n_]^+^, where *n* = 11–23, with H_2_O, together with the complementary reaction involving [Ag^I^(H_2_O)_n_]^+^ (where *n* = 25–45), with NH_3_ using Fourier-transform ion cyclotron resonance (FT-ICR) mass spectrometry. In both systems, it was found that ligand exchange reactions indeed proceeded, and gave rise to “clusters” with only limited numbers of NH_3_ ligands present. Supporting density-functional theory (DFT) calculations demonstrated a competition between Ag(I)'s preference for *sd*-hybridization, and its capacity to cause ligand polarization effects, which influenced hydrogen-bonding strengths, along with the potential of the solvent system involved, to generate extended hydrogen-bonded networks.

Interestingly, one report from 1986 (24) found that the [Ag(NH_3_)_2_]^+^ complex reacts with excess ammonia in aqueous solution to form the triammine derivative {[Ag(NH_3_)_3_]^+^}, with a thermodynamic equilibrium constant of 2.51 × 10^−2^ at 25°C in 0.10 M ammonium chlorate (NH_4_ClO_4_). Therefore, with rather a large excess of NH_3_ present in the “neat” SDF product medium utilised for our investigations (14), there will presumably also be significant quantities of the triammine species present, and this may also influence the reactivity of this agent *in vivo*, and its MoAs, perhaps even markedly so.

Overall, silver(I) generally adopts linear or tetrahedral geometries in its co-ordination complexes, with Ag(I) ions being predominantly two-, three-, and four-coordinated (five- six- and seven-coordinated silver complexes are indeed very rare). The severe lack of Ag(I) complexes with higher coordination numbers arises from the paucity of stereochemical preferences associated with a filled-shell d^10^ electronic configuration.

## 3 SF chemistry—aqueous solution Status and equilibria

Two very unique and distinctive features of the chemistry of 1:1 SF are firstly its ability to form hydrates in aqueous solution and the solid-state [unlike other silver(I)-halide species such as the chloride, bromide and iodide], and secondly its high level of solubility in water, which is reported to be as much as 14.19 mol./L at 25°C (equivalent to a quite considerable 1,800 g/L!). In marked contrast, silver(I)-chloride, -bromide and -iodide are all virtually insoluble in aqueous systems. These properties represent major advantages for the therapeutic applications of SF complexes in the oral health and dentistry fields, since only small volumes of such high concentrations of these agents may be therapeutically-delivered directly and manually to their sites of action by dental surgeons or oral healthcare staff, predominantly at carious dentin tissues for the effective treatment of dental caries, for prophylactic or direct therapeutic purposes, or otherwise. These valuable solubility and hence solution status properties of SF are largely ascribable to the ionic nature of the Ag(I)-F bond, which has been computed to be 70% ionic, whereas those for Ag(I)-Cl, Ag(I)-Br and Ag(I)-I are 30, 23 and only 11% respectively (25). In view of its very high hydration energy (100–110 kcal./mol (26, 27), a value which is similar to that of a C-H covalent bond, the “free” F^−^ ligand has a strong level of solvation when present in aqueous solution media, and this again strongly contributes towards the high solubility of Ag(I)-F. Indeed, F^−^ has a small ionic radius (1.47 Å), and therefore the convergent positioning of multiple “binding” sites for the water molecule H-bonds around this “hard” donor ligand is rendered somewhat complex.

Nevertheless, silver(I) has the ability to form complexes with fluoride ions in aqueous solution. Indeed, in aqueous media at an (extrapolated) ionic strength (I) value of 0.00 mol./L, 25°C and pH values which apparently avoided interferences from HF, an estimate of the equilibrium constant for formation of the 1:1 SF complex was long ago documented to be 2.3 ± 0.2 M^−1^ (Equation 1) (28). However, at an I value of 0.50 mol./L, the equilibrium quotient (C_13_) determined was 0.65 ± 0.05 M^−1^, and this value decreased to 0.55 ± 0.05 M^−1^ at the near-to-physiological temperature of 35°C.


(1) $$\mathrm{Ag}(I)\;+\;F^{-}↔\;[\mathrm{Ag}(I)-F{]}_{\left(\mathrm{aq}.\right)}$$

Concentrated SF clinical product solutions obviously have very high I values, and therefore these may exert a highly significant influence on thermodynamic equilibrium constant determinations. Therefore, if, for the sake of argument, a value of 0.65 M^−1^ is employed for the apparent equilibrium constant (K_app._) for the complexation of Ag(I) by F^−^ in bottles of the SF product just prior to clinical application at ambient temperature, and if any excess F^−^, HF and/or infiltrated HF_2_^−^ levels present therein (14) are ignored, then for an exactly 3.00 mol./L SF product, and a hypothetical neutral pH of the aqueous medium, we can calculate that the concentration of [Ag(I)-F]_(aq.)_ present will be 1.49 mol./L, with “free”, albeit highly water-solvated Ag(I) and F^−^ levels, both being almost equivalent at 1.51 mol./L. These concentrations yield the apparent equilibrium constant (K_app._) of 0.65; HF levels at this pH value are negligible in view of the pK_a_ value for the F^−^/HF system (3.8, Equation 2). The neutral pH parameter assumed here is hypothetical in view of the ready hydrolytic and photolytic decomposition of this product under these conditions (14). Although the authors of the current study accept that this estimate is certainly a little “makeshift”, it was the only one possible in view of the scarcity of thermodynamic equilibrium data on SF complexes, and complications arising from the structural, compositional, pH and excess F^−^ content differences between differing clinical preparations of it, not to mention its ready degradation (especially at pH values >6.0), which generates metallic Ag(0) and Ag(I)-oxide deposits from solution. Complications arising from any excess F^−^, and/or the HF-solvated form of F^−^ (F_2_H^−^) derived therefrom commonly present in these commercial SF products for clinical employment, could arise from the additional direct complexation of Ag(I) by additional F^−^ ligands (as in [Ag(I)F_n_]^(n−1)−^), or by F_2_H^−^ directly {as in [Ag(I)(F_2_H)]}. Therefore, we may conclude that at least some of the Ag(I) ions present in commercially-available SF products for clinical use are in the form of HF-solvated and/or unsolvated Ag(I)-F species, with fluoride coordinated to this metal ion; the rapid ligand-exchange properties of such SF complexes in aqueous solution were readily verifiable by our ^19^F NMR experiments (14). In contrast, human saliva has a much lower I value than that of these “neat” caries-arresting products, and this will undoubtedly affect the thermodynamic equilibrium constant for the formation of Ag(I)-F complexes.

Interestingly, to date it certainly appears that there has been little or no consideration of the solution-based complexation chemistry of SF complexes employed for dental or oral health treatments. In view of this consideration, fluorido-Ag(I) complexes of higher order than 1:1 F^−^:Ag(I) stoichiometry, or concentrated aqueous solutions containing therapeutically-active levels of these agents, serve as major opportunities for future clinical exploitation. Such agents include those with fluoride-exchangeable Ag(I) complexes, which are detectable in our ^19^F NMR studies. In principle, higher-order and/or pH-dependent [Ag(I)(F_2_H)] complexes present in SF products may have the ability to drive and deliver “excess” fluoride to active disease sites on a mole-for-mole basis, since each Ag(I) ion carries and may convey more than a single fluoride ligand. Significant levels of the Ag(I)F_2_H complex present in acidotic physiological media [Equations 2 and 3, where the dotted line between the Ag(I) and F^−^ ions represents rapidly-exchanging F^−^ at the Ag(I) centre], such as those of carious dentine lesions (29), may also offer some additional therapeutic benefits, and these require further attention, including detailed laboratory-based investigations focused on its MoAs.


(2) $${H^{+}\;+\;F}^{-}↔\;\mathrm{HF}\;(pK_{a}\;=\;3.8)$$

(3) $$Ag^{I\ldots }F^{-}\;+\;\mathrm{HF}\;\to \;[Ag^{I}(F_{2}H)]$$

**Figure 1:**
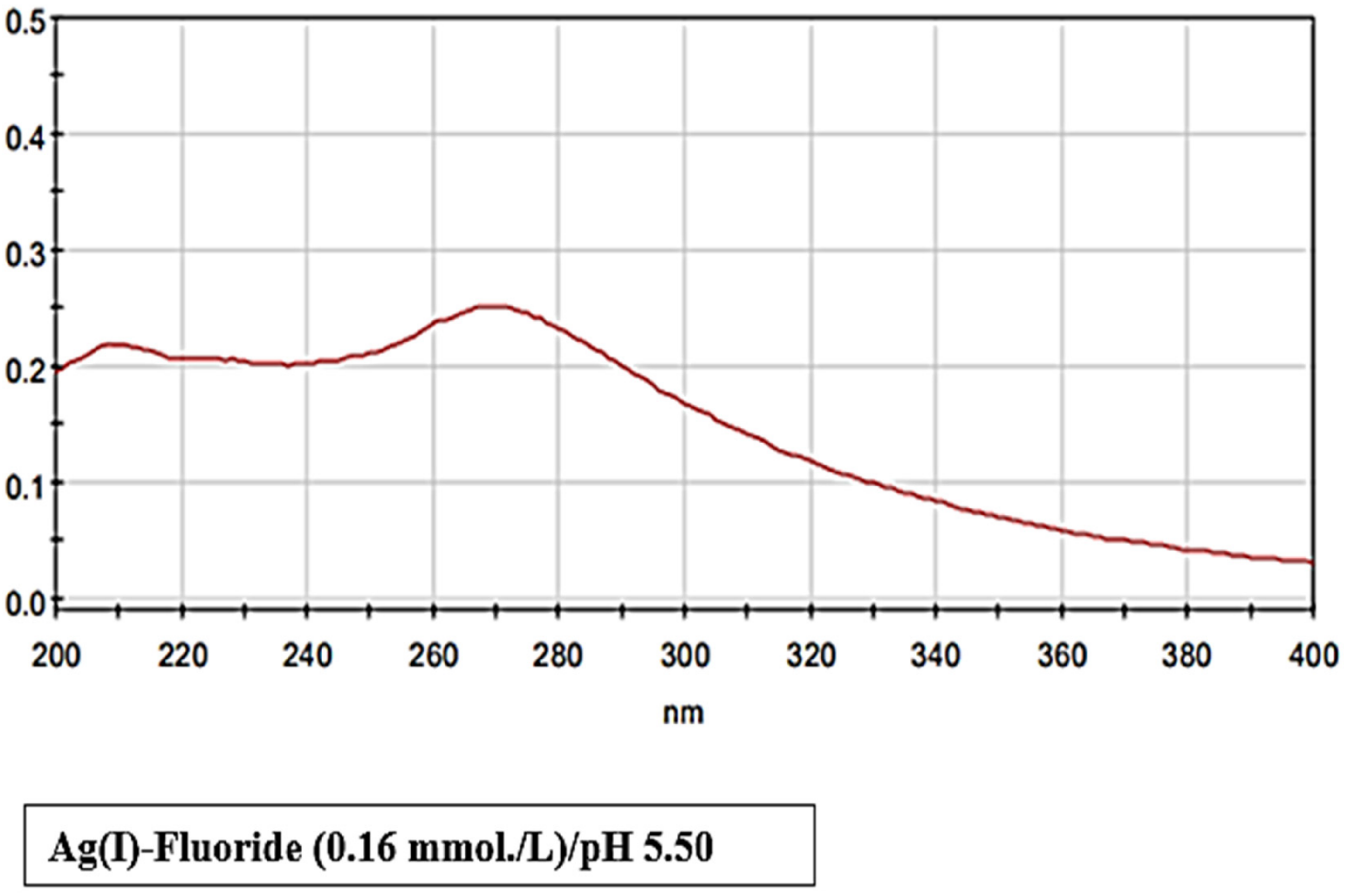
Electronic absorption spectrum of a solution containing a commercial sample of 1:1 SF (0.16 mmol/L, pH 5.50) available for synthetic chemistry, and not clinical dental applications, in HPLC-grade water.

Notably, most commercial sources for the purchase of SF products for investigations of their chemistry and chemical reactivity [notably, their use as fluorinating agents in synthetic organic chemistry (25)], have been found to be acceptable for these purposes. However, although this purified agent should be yellow/orange in colour, which presumably arises from a ligand-to-metal charge-transfer absorption band in the near UV-region centred at *ca.* 270 nm (Figure 1), this colouration may also indicate some trace contamination by bronze- and brown-coloured silver sub-fluoride [Ag^I^_2_F] and silver difluoride [Ag^II^F_2_] species respectively (25). In view of this consideration, some caution should be applied to the interpretation of the spectrum shown in Figure 1, since some of the absorbance noted in the 200–400 nm region may arise from these trace contaminants with differential silver oxidation states. Contrastingly, certain scientific literature information available has stated that whereas the anhydrous form of Ag(I)-fluoride is actually coloured, its various hydrates, apparently, are not (30). However, that was a reference from 1954! These hydrates, Ag(I)F.(H_2_O)_2_ and Ag(I)F.(H_2_O)_4_, are reportedly formed on precipitation from aqueous solution.

A further recent study, however, explored the use of Ag(I)-fluoride for the fluorodesulfurisation of thionobenzodioxoles (31). For this purpose, the researchers involved conducted a systematic examination of reaction variables, and concluded that the commercially-available source of Ag(I)-fluoride utilised for their experiments (which was originally brown-to-orange in colouration) gave rise to a black insoluble precipitate on attempted dissolution in water. This observation presumably arises from the deposition of dark brown/black silver(I)-oxide and metallic silver from hydrolysis of the Ag(I) ion at neutral or near-neutral pH values, which we commonly observe in the laboratory, although at the mildly acidic pH values used in Ref (14). and the current study, or lower ones, Ag(I)-fluoride remains stable in aqueous solution. Some finely-divided metallic Ag(0) arising from photoreduction of Ag(I) may also be present in this precipitate. The absorption spectrum shown in Figure 1 was obtained at a pH value of 5.5 which was found to be sufficient for the stabilization of this agent; solutions prepared at pH 7.0 were, however, quite rapidly transformed to a black deposit following preparation, and were very light-sensitive [Figure 2 in Ref (14)]. This spectrum shows absorption maxima located at 208 and 269 nm, the latter of which is presumably a ligand→metal ion charge-transfer band, and stretches into the near-visible region of the electromagnetic spectrum, and hence is responsible for the yellow colouration of this complex.

**Figure 2:**
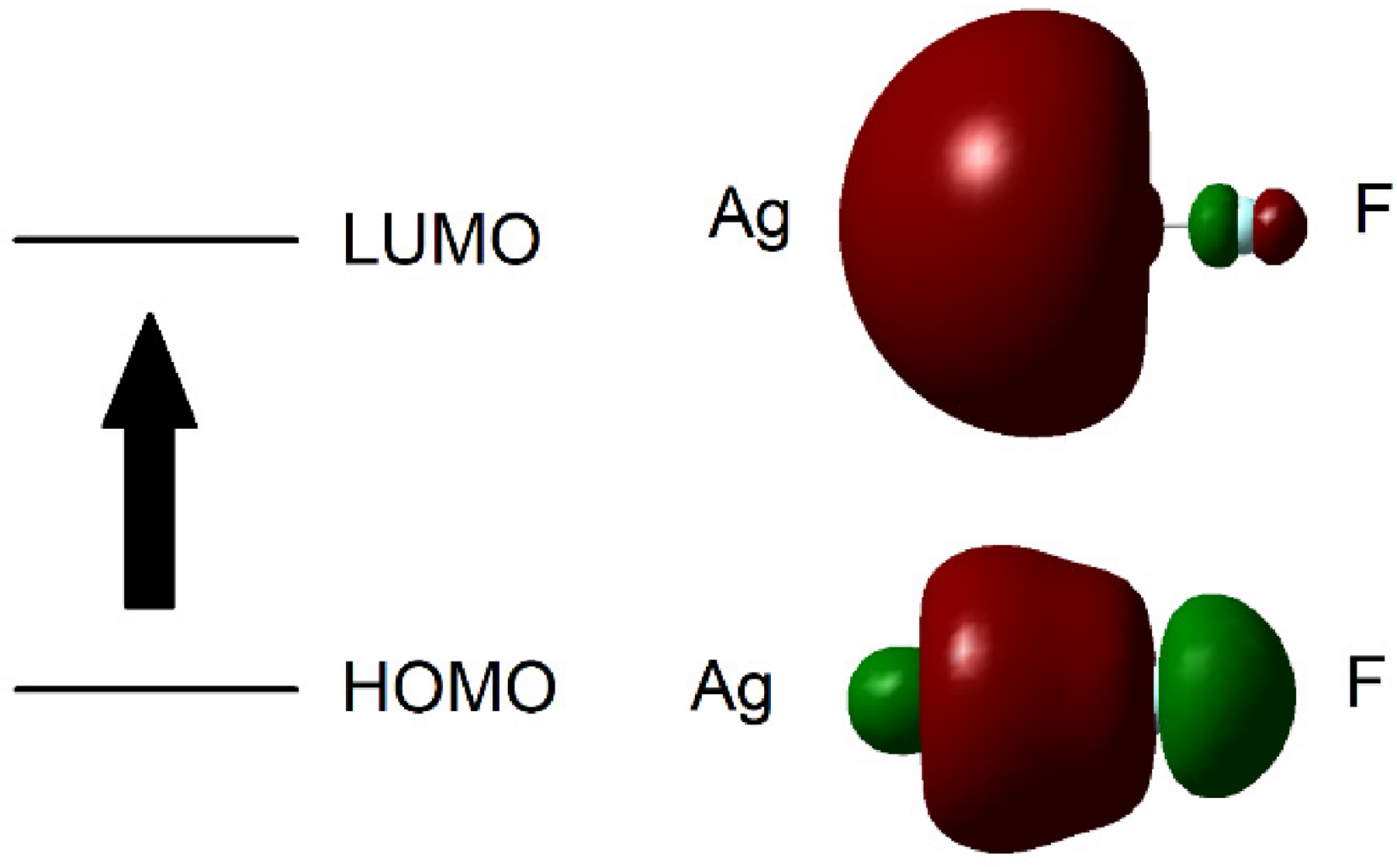
Schematic representation of the calculated HOMO and LUMO molecular orbitals for Ag-F. The arrow indicates the calculated transition between these orbitals.

In Ref (31), the authors continue with the statement “To remedy this, the commercial AgF was dissolved in water, filtered through a cotton plug, and concentrated *in vacuo* (protected from light), which provided silver(I) fluoride as a light yellow solid; the insoluble black solid remains on the filter.”

Unfortunately, there are little or no reports available on the electronic absorption spectrum of Ag(I)-fluoride, most especially in aqueous solution, possibly because of problems with obtaining this material as pure, and free of any UV- or visible region-absorbing contaminants. However, one publication from 1968 (32) reports on its spectrum in the gaseous state, and this found an absorption maximum at 303 nm. Further bands were also observed at lower energy, and also at <260 nm.

### 3.1 Gaussian-03 excited state calculations for the 1:1 [Ag(I)-F] complex: simulation of electronic absorption spectra *in vacuo* and in aqueous solution

These excited state and absorption spectrum simulation computations were performed using Revision B.03 of the Gaussian 03 software module (Frisch et al., Gaussian, Inc., Pittsburgh PA, 2003). The output files from Gaussian excited states calculations reported the excitation energies and oscillator strengths for each excited state transition. Configuration Interaction (CIS) and time-dependent (TD) excited states calculated the energy required to promote an electron from an occupied to an unoccupied molecular orbital, and the probability of this occurring.

The Ag(I)-F bond length was optimised *in vacuo* using the HF/3-21G, SDD and Lan2DZ methods, and the Self-Consistent Reaction Field (SCRF) solvation model was applied for water, employing the default Polarizable Continuum Model (PCM), with the Integral Equation Formalism Variant (IEFPCM) strategies (Table 1). Details of the calculated energies required to excite an electron from the HOMO to LUMO states in Ag(I)-F are provided in Table 1.

**Table 1:** Calculated energies (nm) required to excite an electron from the highest occupied to the lowest unoccupied molecular orbitals (HOMO to LUMO respectively) in Ag(I)-F.

Method	State	*In Vacuo* (1.92 Å)	SCRF water (2.1327 Å)	15 water (2.395 Å)^a^
HF/3-21G	CIS	283.15	231.66	289.9
HF/3-21G	TD	287.03	235.15	296.03
SDD	TD	267.58	238.80	257.84
Lan2DZ	CIS	258	238.8	255.67
Lan2DZ	TD	261.69	231.11	260.16

CIS and TD, configuration interaction and time-dependent excited states respectively; HF, Hartree-Fock; SDD, dunning/Huzinaga full double zeta (D95) up to element Ar, followed by Stuttgart/Dresden Effective Core Potentials applied to the remainder of the periodic table; Lan2DZ, D95 V applied to the first row of the periodic table [Dunning/Huzinaga valence double-zeta], and Los Alamos Effective Core Potential with Double Zeta on Na-La.

^a^ Indicates the water solvation model with each bonded SF molecule surrounded and hydrated by a total of 15 water molecules.

An optimised bond length computationally determined for the Ag(I)-F system *in vacuo* was 1.92 Ǻ, a value which increased to 2.1327 Ǻ in the solvation model. In this solvation model, a Ag-F molecule was surrounded by up to 15 discrete water molecules, and all atomic positions were optimised, which gave rise to a Ag(I)-F bond length of 2.395 Ǻ. This value was found to be very close to the reported experimental x-ray bond length of 2.46085 Ǻ (33).

From these computations, the average calculated value for the Ag(I)-F ligand-to-metal ion charge-transfer band for the *n* = 15 water hydration model was 271.655 nm, a value very close to our lowest energy absorption band centred at 269 nm (Table 1). A schematic representation of the computed HOMO and LUMO molecular orbitals for Ag-F is shown in Figure 2.

Interestingly, Craig and Brooker (34) performed calculations for hydrated fluoride anion [F^−^(H_2_O)_n_] alone, where *n* = 1–10, at the RHF/6-31 + G* level, and a relatively stable geometry was found for *n* = 6. With *n* > 7, however, evidence was presented for the involvement of further water molecules hydrogen-bonding to the water of hydrated fluoride species. On the basis of these computations, together with *ab initio* calculations or experimental diffraction studies, the mean H_2_O coordination number for fluoride was *n* = 6.

As noted in Section 4.3 of Ref (14), some commercial formulations of both SF and SDF have previously been shown to contain excessive levels of fluoride (35, 36). Moreover, selected researchers have suggested that one or more SF products contain silver(II)-fluoride [Ag(II)F_2_] rather than SF (36), which has an expected molar [F^−^]:[Ag(I)] concentration ratio of 2:1. However, for the products examined our report, the presence of Ag(II) in commercial SF products for clinical use remains completely unfeasible in view of the powerful oxidising actions of Ag(II), and it can readily oxidise water (the Ag(II)/Ag(I) redox potential is close to +2.0 V).

## 4 Rationales for the biological chemistry, disposition and fates of SDF, SF and silver(I)-nitrate in the oral environment: relevance to their therapeutic activities and MoAs

### 4.1 Solution-, solid-, mixed-state and dissolution biomolecular chemistries

For the Ag(I) ion to take part in the essential, differential classes of coordination chemistry featured in its MoAs for caries arrest, and its biological chemistry in general, it will undoubtedly undergo ligand-exchange reactions featuring the release of one or both of its two near-linearly-coordinated ammonia ligands, in addition to the solubility-driven deposition of AgCl, as demonstrated in Ref (14). Indeed, this process will involve the substitution of one or more, but presumably both monodentate NH_3_ donors with powerful endogenous bioavailable ligands (e.g., phosphate or thiocyanate anion). Notably, [Ag(I)-SCN] complexes, such as those potentially observed in our Ag/AgCl nanoparticulate deposits, are polymeric with a zig-zag structure in view of an sp^3^-hybrized sulphur atom (37). Because many low-molecular-mass chelating ligands are unable to form linear complexes with Ag(I) in view of their limited molecular geometry and dimensions, they also tend to form polymeric species. However, in principle, Ag(I) still has the ability to cross-link proteins or even larger peptides, either intra- or intermolecularly.

Although some recent clinical trials are now or have recently been exploring the combined use of silver(I) complexes, or admixtures of these with fluoride varnishes semi-annually for the treatment and arrest of dental caries in young human populations, to date it certainly appears that there has been little or no consideration of the complexation chemistries of SDF and SF complexes, nor that of the relevant or opportunistic silver(I) or (0) biochemistry options considered for such studies. Such species will include those with F^−^-exchangeable Ag(I) complexes, particularly those detectable in our ^19^F NMR studies performed on commercial SF products in aqueous solution [Figure 1 in Ref (14)].

In 2020, Alharbi and Almugren (38) conducted an overview and systematic review of the indications for use, effectiveness and disadvantages of SDF as a therapy in paediatric dentistry through relevant literature searches of published material up to May 2018. From this investigation, they deduced that the simplest, most efficient, and least costly preventative approaches were anti-bacterial silver(I)-nitrate and cariostatic fluoride anion, and that SDF represented a composite of these two types of therapy. Indeed, Ag(I) ion acts as a highly effective bactericidal agent, and when this is closely associated with tooth structure, opportunities to inhibit bacterial attachment, reproduction, and biofilm formation are created.

Hence, it is generally accepted that SDF acts via two synergistic mechanisms in order to protect healthy tooth structures against bacterial-mediated caries development: Firstly, to facilitate the creation of fluorohydroxyapatite crystals which are less soluble than hydroxyapatite under physiologically-low pH conditions, and secondly to create a hostile environment for bacterial metabolism and biofilm formation through the actions of the central Ag(I) ion, which is also taken up by this biomineral. In early studies based on examinations of the modes of action of sodium fluoride and silver(I)-nitrate on teeth, investigators found that complex MoAs are involved for both agents (39, 40), as expected. As noted above, the most commonly recognized, albeit historical, interaction is that of sodium fluoride with hydroxyapatite to form fluorohydroxyapatite (Equation 4, which is only a simplified schematic representation of a very complex process), although a less commonly acknowledged reaction involves the combination of tooth Ca^2+^ with F^−^ anion to form virtually insoluble CaF_2_. The primary reactions of silver(I)-nitrate involve the formation of silver(I)-phosphate and silver(I)-oxide [Ag(I)-phosphate and Ag_2_O respectively], although insoluble AgCl will also form in the presence of chloride. Any change in medium pH from these reactions is presumably counteracted via neutralisation with the quite strong buffering capacities of saliva, or other oral fluids and tissues which have access to its site of generation.


(4) $$Ca_{10}(PO_{4}{)}_{6}(\mathrm{OH}{)}_{2}\;+\;2F^{\text{-}}\to \;Ca_{10}(PO_{4}{)}_{6}F_{2}\;+\;2OH^{-}$$

Interestingly, the detection of phosphate species in the Ag/AgCl nanoparticulate deposits formed in human saliva following SDF addition to it by FTIR-ATR and ^31^P NMR spectroscopic analyses (14), indicates that such samples may also contain some Ag(I)-phosphate complexes. ^31^P NMR analysis confirmed the presence of inorganic phosphate in the WMSS samples evaluated.

*In vitro* studies have indicated that SDF penetrates enamel to a depth of 25 microns, and approximately 2–3 times more fluoride is retained than that delivered by sodium monofluorophosphate, sodium fluoride or tin(II)-fluoride (41), so this certainly indicates that [Ag(NH_3_)_2_]^+^ ions, and/or its Ag(I) ion biotransformation products, may act as fluoride delivery agents. The demineralization of surface enamel arising from the low pH conditions produced by dietary sugar-fueled oral biofilm metabolism will enlarge its surface area, and hence modify the bioavailability of structures upon which SDF or its derivatives may act upon or react with.

**Figure 3:**
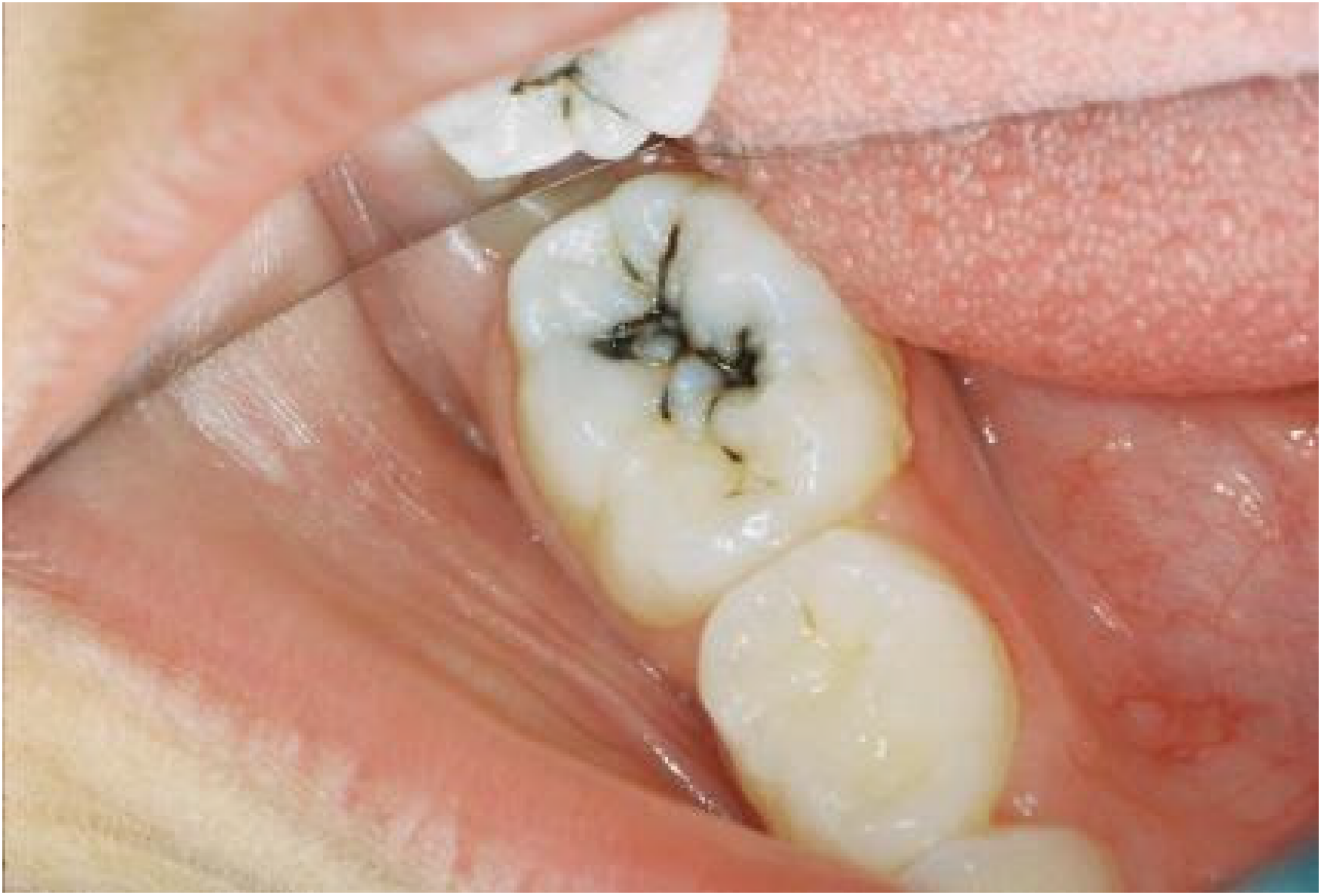
Photograph of SDF-induced black staining present in a demineralized enamel fisssure. (Photo: Courtesy of S. Duffin—informed consent to show this photograph here was obtained from the patient involved).

To date, hypothesized biochemical reactions which lead to the dark/blackened metallic silver base discolourations of treated surfaces appear to involve the primary formation of Ag(I)-phosphate in demineralized enamel and dentin. A healthy enamel surface will allow the diffusion of silver(I) ions into the interprismatic space, but will not react with it to engender any discoloration. This is opposed to demineralized enamel, which indeed does so, the stain perceivably arising from the hydrolysis and/or photodegradation of Ag(I)-phosphate. However, many alternative Ag(I)-biomolecule complexes may also degrade in this manner, and it is this feature of SDF's biological actions which may be considered to serve as a caries detection system (Figure 3).

However, it should be noted from our examinations of the interactions of SDF, SF and silver(I)-nitrate with human saliva that insoluble AgCl initially generated from these processes may be readily removed and transferred elsewhere following its deposition on healthy enamel surfaces through motional actions of the tongue, and that this activity is likely to occur prior to its reduction and/or photoreduction to CSNPs. However, this is much less likely to be the case when it is deposited within dentin, or porous demineralized enamel, this “sheltering” process providing sufficient time for these Ag(I) complexes to be transformed to Ag(0)-containing nanoparticles through the actions of salivary or other oral fluid electron-donors, and/or much less likely photosynthetic processes. Of much importance, the former mechanism is more probable in view of the potentially limited exposure of available Ag(I) to light within the oral environment. Nevertheless, such nanoparticulates generated from the salivary-mediated IV-SCAN process can be deposited in the crevices or fissures of demineralized enamel, and at such sites these species may then be degraded to black-coloured Ag(0). Rather than arising from the reaction of Ag(I) with hydroxyapatite to form insoluble Ag(I)-phosphate complexes, this clearly provides an alternative, and perhaps more physiologically-relevant explanation for the black discolourations formed and observed *in vivo* following SDF treatment, or indeed that found with other silver(I) complexes such as SF or Ag(I)-nitrate.

Notably, some evidence has indicated that the largely-insoluble CaF_2_ produced from the interaction of Ca^2+^ with SDF- or SF-derived fluoride may be responsible for the strengthening of carious dentin. Indeed, it may be deposited within dentin tubules and therefore successfully block them as an insoluble matrix, so that this complex serves as a desensitizing agent. Furthermore, this process also appears to provide an environment which promotes fluorohydroxyapatite generation, although Lou et al. (42) have proposed that CaF_2_ formed in their experiments was washed away from this material. However, significant amounts of this solid may also be generated from the direct interaction of salivary Ca^2+^ with added F^−^.

Historically, to date only a limited number of reports are available on the modes of action of SDF towards mineralized tissues, however. Although in 1972 Yamaga et al. (40) originally proposed that the *in vivo* generation of both CaF_2_ and Ag(I)-phosphate complexes may account for the arrest of dental caries and the hardening of carious lesions, a little later Suzuki et al. (41) confirmed the production of CaF_2_ by equilibrating/mixing SDF solution with an enamel powder, although it is important to note that the quantity of CaF_2_ formed diminished markedly so when these agents were submerged into an artificial saliva preparation. Similarly, these researchers also discovered that Ag(I)-phosphate was removed following immersion in this artificial biofluid, and that both solid AgCl and a 1:1 silver(I)-thiocyanato complex [the latter with a polymeric structure (37)] were generated from SDF treatment, results similar to those observed in our studies focused on the interactions of SDF with human saliva (14). More recently, Lou et al. (2011) (42) found that a globular material resembling CaF_2_, along with metallic silver, were produced on the surface of hydroxyapatite by the introduction of SDF solution to an admixture containing hydroxyapatite powder and gelatine, the latter serving as a “typical” biorepresentative protein, which unfortunately it is not, although in view of its very high proline, hydroxyproline and glycine contents, it certainly is for collagen, as we might expect. However, the “CaF_2_-like” material was found to dissolve and was therefore removed following a water washing process. Therefore, the ultimate mode(s) of SDF action remained somewhat unclear from these results. However, the solubility of CaF_2_ is reported to be 1.6 mg/L at 20°C, equivalent to 20.5 µmol./L, which may be considered to be quite significant in at least some physiological contexts. Notwithstanding, these results are fully consistent with our results concerning the removal of significant levels of SCN^−^ anion from WMSS samples on treatment with SDF (as monitored by FTIR-ATR spectroscopy), and its deposition in the Ag/AgCl nanoparticulate deposit formed therefrom (14); this may indicate its co-complexation by Ag(I).

Lou et al. (42) also found that Ag(I)-nitrate yielded yellow-coloured cubic crystals of Ag(I)-phosphate, which were not dissolved on washing, decolourised and darkened, and transformed to Ag(0) on light exposure. These researchers also found that with gelatin, SDF and Ag(I)-nitrate formed wash-resistant metallic silver particles, whereas as expected, F^−^ exerted no overall macrochemical effects. Accordingly, they concluded that the “solubility” of their putative CaF_2_ material observed appears to negate against its caries-protecting role since it will not be retained thereon for very long durations, and therefore they concluded that further investigations were required in order to study the importance of the so-called “persistent” silver species or materials observed in their experiments.

In 2019, Peng et al. (43) explored the dose-response behaviour of SDF when equilibrated with demineralized dentine in a basal medium mucin (BMM) salivary substitute, and also unstimulated human saliva (USS) samples. For this purpose, dentine discs, which were stored in these media, along with a salivary substitute, were then chemically demineralized, and increasing concentrations of SDF [0.01 ml aliquots of 10, 24 or 38 (w/v) % SDF] were then added; the mixtures were subsequently stored for a duration of 5 days. Following this period, the dentine disc samples were digested in 70% (w/v) nitric acid, and silver was determined by an ICP-MS technique. These researchers found that the dentine silver content increased proportionately with increasing added SDP level for both the BMM and UWS groups. However, at added concentrations of 10 and 38 (w/v)%, the BMM group gave rise to significantly higher silver levels than the USS one, and the authors noted that SDF interacts more so with BMM, and with a higher level of silver deposition, than that found using USS. One clear conclusion from this work was that the prognostication of *in vitro* studies conducted with artificial saliva for the evaluation of interactions between SDF and tooth matrices should be viewed with at least some caution. The authors of the current paper thoroughly agree with this point—substitutes for “real” human saliva are actually very poor for such bioinorganic chemistry studies, most especially since they certainly lack many critical metal ion-complexing biomolecules such as amino acids and organic acid anions, the latter of which are present in this medium at levels sometimes exceeding 1–10 mmol/L, for example lactate and acetate (29).

Recently, SDF, coupled with or without the post-application of potassium iodide (KI) for the subsequent stigmatising stain removal, featured as the only therapy employed in the Healthy Kids Cambodia project (44). However, this project erroneously only mentioned “silver fluoride solutions” as the therapeutic agent evaluated in its title, and not SDF. In view of the above evidence provided, SDF effectively blocks demineralization and strongly facilitates the remineralization process. In general, SDF treatment of arrested carious lesions generally gives rise to a black and hard surface. This common clinically-observed phenomenon is further supported by evidence obtained from an *ex-vivo* investigation which found an enhancement in dentine surface layer microhardness following its application (45). Moreover, corresponding laboratory investigations discovered that dentine carious lesions had a surface layer which was rich in Ca^2+^ and phosphate ions subsequent to treatment with SDF (45, 46). Of especial interest, human saliva represents a medium which is “supersaturated” with these ions, and therefore this biofluid affords the remineralization of teeth in the presence of SDF at pH values > 5.5 (47). Such observations may therefore involve the products identified from the reactions of SDF with this biofluid in the study reported in (14), including novel CSNPs, in addition to F^−^ anion, both “free” and Ag(I)-exchangeable.

In 2002, Wu and Yang (48) also found that SDF had the ability to retard enamel and dentine demineralization, in addition to its role in suppressing hydroxyapatite degradation through the fluoridation of this biomineral. Following SDF application, one additional investigation (49) revealed that enamel carious lesions lost significantly lower quantities of minerals when compared to those observed without applying this treatment option, and that tooth surface plaque absorbed fluoride. During short-chain organic acid production from bacterial catabolism, plaque fluid F^−^ was found to be able to infiltrate the enamel sub-surface, and this active agent was then assimilated onto the crystal surface, a process which protected it against dissolution (50). Apparently, insoluble silver(I)-protein complexes are generated, together with CaF_2_ and Ag(I)-phosphate, and all these precipitates were found to be deposited on the dentine surface following SDF treatment (51). However, dense granular structures of spherical grains located on dentine intertubules were developed from such precipitates, and these obstructed dentinal tubule orifices (52). It was also found that precipitates with high Ca^2+^ and phosphorous contents actually diminished the loss of both these species from carious dentine lesions. Despite the presence of Ag(I)-phosphate in such precipitates, this insoluble complex is attacked by chloride anion, a ligand which readily substitutes for phosphate (53), since the solubility product of AgCl is lower than that of Ag(I)-phosphate (54); this represents the thermodynamic driving force for this reaction system.

Again, these observations are of much relevance to our study described in Ref (14), since we found that partially-discoloured AgCl is primarily generated from the interactions of SDF, SF and Ag(I)-nitrate to human saliva, a process which precedes the time-dependent evolution of CSNPs. Overall, scientific literature evidence available confirms that once formed, Ag(I)-phosphate is converted to more insoluble AgCl in the first instance (52, 53), and therefore when localized and “protected” by the crevices and fissures of demineralizing enamel, coloured nanoparticulate materials will have sufficient time to develop therefrom, followed by the terminal deposition of dark-coloured metallic silver from this source.

Intriguingly, SDF also appears to have the ability to remineralise hypomineralised teeth, this representing a developmental disorder which customarily affects first molars and permanent incisors (55). Indeed, white- to dark-brown-coloured, well-defined opacities typically characterize clinically-hypomineralised teeth (54); these opacities are histologically porous, and are positioned within the inner region of enamel. Severe hypersensitivity represents a frequent problem in children with hypomineralised teeth in view of rapidly progressing tooth wear, and the exposure of dentine. These patients are also particularly susceptible to dental caries in view of flawed enamel structures inherent to their condition. However, SDF has been found to act as a relatively simple, pain-free and overall non-invasive treatment option for the purpose of counteracting this disorder (56). Indeed, the use of SDF may effectively desensitize such hypomineralised teeth, and also rapidly halt the further development of carious lesions thereon. Moreover, one clinically-based investigation discovered that biannual SDF therapy circumvented caries progression and diminished dentine hypersensitivity in hypomineralised molars (56). This study therefore concluded that repeated applications of SDF on hypomineralised teeth offered a substantial relief of dentine hypersensitivity, this effect having a high level of longevity.

In Ref (53), Mei et al. confirmed that SDF facilitated the remineralization and hardening of carious lesions. Prior to this report appearing, the actions of SDF towards hydroxyapatite (HA) crystallization were not strictly clear, and therefore researchers involved in this project performed *in vitro* investigations involving the equilibration of calcium phosphate with increasing SDF concentrations (0.38–3.80 g/L) in order to explore its effects towards the nucleation and growth of apatite crystals (appropriate control groups containing calcium chloride plus potassium hydrogen phosphate in buffer solution, and SDF alone in buffer solution, were also evaluated). This extensive study then featured the applications of a wide range of analytical and crystallographic techniques, including crystal unit cell parameters monitored by powder x-ray diffraction, and assessments of their shape and organization by bright-field transmission electron microscopic (TEM) techniques. Also included were studies of the rotational and vibrational status of crystal phosphate functions by Raman microscopy. Both TEM and selected-area electron diffraction demonstrated that all precipitated solids obtained from the SDF-treated groups were indeed crystalline, with a positive correlation being found between added SDF levels and increases in the crystal size proportion. Moreover, powder x-ray diffraction patterns provided evidence that both fluorohydroxyapatite, and precipitated AgCl, were formed in these SDF treatment groups. Notably, a higher field shift of the Raman spectrum phosphate function band was observed in all the SDF-treated groups, and this was consistent with an a-direction unit cell contraction, which was not observed in the c-direction in these groups when compared against the calcium phosphate control group. These results indicated the substitution of hydroxyapatite hydroxide by small localised fluoride ions. Overall, the observations made from these experiments suggested that the reaction of SDF involved modifications in the phosphate groups of hydroxyapatite, processes which gave rise to fluorohydroxyapatite. In view of its limited water-solubility, again the deposition of fluorohydroxyapatite was considered to be largely responsible for the arrest of caries lesions induced by SDF treatment.

In addition to the influence of F^−^ on the mineral and organic acid status, and constitution of dentine, silver(I) ions may also directly contribute to the arrest of dentine caries development, notably by exerting their killing effects towards pathogenic bacteria, as reviewed in (54). Indeed, Ag(I) ions are known to damage both bacterial cell membranes and bacterial enzymes, and these factors clearly block bacterial growth and preponderance. This metal ion can also be incorporated into hydroxyapatite, and can exert an antibacterial effect at this site; indeed, silver(I) ion-complexed hydroxyapatite can also exert quite powerful bactericidal actions, and therefore protects dental hard tissues against acid-induced damage, and sequentially the development of dental caries. Moreover, Ag(I) ions are also powerful inhibitors of cathepsins, and in view of their affinity for Ag(I) complexation, or even its potential macromolecular cross-linking capacity, amino acids present in collagen, particularly its critical cysteine thiol residue, prevents the degradation of this pathologically-key biomacromolecule (52, 57, 58). Although earlier studies originally hypothesized that Ag(I) ions in SDF-hardened caries lesions act through the generation of Ag(I)-phosphate complex species, as noted above, more recent investigations have revealed that only a small quantity of Ag(I)-phosphate remained on the arrested lesion. Indeed, the major silver-containing precipitate formed was actually AgCl, its generation being driven by its high thermodynamic stability constant and very low solubility product (59), and which was not at that time conceivably involved in the significantly enhanced hardening of arrested dentine lesions. However, fluoride anion-promoted mineralization occurs via the formation of fluorohydroxyapatite, which has a health-promoting diminished solubility. Increased contents of both Ca^2+^ and phosphate, but not of silver, on the surface layer of arrested caries lesions was found following treatment with SDF, and this gave rise to significant enhancements in microhardness. Interestingly, fluoride ion also has the capacity to suppress the activities of matrix metalloproteinases, and because of this, it also blocks the deterioration of dentine collagen. Furthermore, in an alkaline medium, such as that observed in nearly all commercially-available solution formulations of SDF, it appears that the therapeutic, if not chemical, combination, of Ag(I) with F^−^ act in a synergistic capacity towards the arrest of dental caries, with the effect of SDF's alkaline medium affording an unfavourably high pH matrix for the growth and preponderance of cariogenic micro-organisms, a process which circumvents the advancement of carious lesions. As previously stressed, a full understanding of the mechanisms involved in this process will serve to facilitate the development of approaches for the clinical applications of SDF and SF for the treatment of caries-susceptible populations.

These results obtained in demineralized enamel and dentine are again fully consistent with our observations of AgCl deposition in clear human salivary supernatants arising from SDF addition, followed by the time-dependent development of probably therapeutically-active CSNPs on particulate AgCl reduction or photodegradation (14). As noted above, these demineralised hard tissue sites, with inherent fissures and crevices, are presumably required to retain sufficient AgCl deposits as sources for the production of potentially therapeutically-active CSNPs in a time-dependent manner.

### 4.2 SF- and silver(I) nitrate-based alternatives to the relatively expensive SDF treatment available for caries arrest

Since SDF can be considered to be more expensive than SF in view of its requirement for carefully-planned synthesis involving the additional treatment of Ag(I) ions or Ag(I)-F species with two molar equivalents of ammonia, SF may serve to offer a less costly alternative for the treatment of dental caries patients in poor communities and countries with only limited or even a negligible availability of dental clinicians and further oral healthcare staff. Therefore, there is currently some research focus on the provision of these cheaper alternatives, and these include established or known SF products, along with nanoparticulates containing them or derived therefrom.

A two-stage combined topical therapeutic strategy, specifically that involving SF followed by tin(II)-fluoride (SnF_2_) therapies, was employed by Green (10) as a caries-preventative regimen for children, and was applied to the treatment of newly-erupted first molars. For comparative purposes, a second (control) group of children received the subsequent topical SnF_2_ therapy only. Treated teeth in all patients were monitored for an 18-month duration at 6-month intervals. For this study, the author found and concluded that when teeth received the SnF_2_ treatment only, a significantly greater caries development incidence was observed, data consistent with the valuable therapeutic effects offered by SF.

In 1989, Gotjamanos (11) histologically examined the dental pulps of carious primary teeth (*n* = 55) at variable periods of 3–58 months following their treatment with 40% (w/v) SF via an “atraumatic” application to any remaining caries residues. This approach was followed by a glass ionomer cement restoration. Histological comparisons with treatments not involving SF but those featuring restorations made using glass ionomer cement, zinc oxide and eugenol, or amalgams, found that the great majority (50/55) of the teeth investigated yielded a favourable SF-mediated pulpal outcome, which gave rise to an abundance of reparative dentine, along with a broad odontoblast layer. However, the author of this report concluded that the use of pulpal histology alone could not provide sufficient evidence that SF treatment serves as an effective and acceptable clinical strategy for the arrest of residual caries. It was also concluded that the excessive levels of F^−^ present in commercially-available SF products may represent a significant toxicological risk factor for patients.

Intriguingly, a further study (12) involved a comparative evaluation of the caries lesion-arresting properties of SF and SDF in children, both with and without follow-up treatment with application of potassium iodide as a dark/black stain-removing step according to stage two of the manufacturer's recommended protocol. For this purpose, a four-armed parallel design cluster-randomised controlled trial experimental design was employed, and four protocols for caries arrest at 6 m and 12 m were examined. The influence of the post-SF and -SDF application potassium iodide (KI) treatment on lesion colouration was also assessed. With the use of a two-level logistic regression modelling system, this investigation found that treatment with both these Ag(I)-containing products yielded high caries arrest rates (75% and 77% for SF and SDF respectively). However, when coupled with post-application of the KI treatment, these arrest rates decreased to only 65% and 51% respectively, and this adverse colouration-diminishing application had a lower odds ratio of caries arrest. However, as expected, the groups receiving KI had an associated significantly higher odds of circumventing such black discolourations. Therefore, the researchers involved concluded that the employment of KI is significantly related to a lower level of caries arrest and management, but with a much improved aesthetic benefit.

Overall, a consideration of the bioinorganic chemistry involved in the post-Ag(I) complex application of iodide anion (I^−^), which precipitates one of its key agents [microbicidal Ag(I) ions] as insoluble silver(I)-iodide [Ag(I)-I], which is actually yellow/pale-yellow in colouration, is likely to exert a deleterious negative effect regarding the caries-arresting properties of both SF and SDF. Unfortunately Ag(I)-iodide is also photosensitive, and therefore it degrades to metallic Ag(0) with time; indeed, metallic grey colourations can be viewed in the solid material, especially when it is exposed to light. Interestingly, insoluble silver(I)-iodide can be rendered more water-soluble in the presence of excess iodide, where higher, more soluble complex species such as [Ag(I)I_2_]^−^ may be generated [original study published in 1952 (60)], as is also the case for insoluble Ag(I)-chloride and -bromide when treated with excess chloride and bromide respectively. Hence, addition of an appropriate excess of I^−^ to the treatment site, so that there is a large excess of it over that of insoluble Ag(I), will not only decolourise the black stain, but will also facilitate solubilisation of the induced pale-yellow AgI solid formed.

A quite recent randomized clinical trial performed by Gao et al. (2019) (61) revealed that a biannual application of 25% (w/v) Ag(I)-nitrate solution followed by a 5% (w/v) fluoride anion-containing varnish was described as “no worse than a 38% (w/v) SDF solution” in arresting dental caries amongst pre-school children over 18 months of age. Since this dual treatment regimen was non-invasive and very simple to apply, it served as an alternative strategy for the successful management of dental caries in young children, most notably in countries where SDF is unavailable or too costly to apply for poverty-stricken populations. This report demonstrates that cheaper, more readily-available alternatives to SDF treatment are, in principle, achievable for third-world countries. Indeed, it is expected that this study will spark a renewed interest in the use of this more basic treatment as a cheaper alternative to SDF. Although SDF treatment regimens are relatively inexpensive when compared to those of more traditional Western world ones, even cheaper alternatives are sought for widespread use in third-world populations, predominantly paediatric ones.

Intriguingly, it is conceivable that the above dual function treatment gives rise to the generation of solution-state fluorido-Ag(I) complexes from chemical reactions engendered from the sequential addition of the fluoride varnish subsequent to Ag(I)-NO_3_ application, quite possibly those with the Ag(I) metal ion centre exchanging with more than a single coordinating fluorido ligand, as indicated in (14). However, this will, of course, depend on the retention of phase-1 treatment Ag(I) at active sites during the phase-2 F^−^ varnish application. Despite their clinical efficacies, fluoride varnish products remain expensive, the application technique involved for their use is sensitive, and multiple applications of them are required per annum. Consequently, alternative formulations that provide these and further benefits will be of especial interest to the dental community.

Atraumatic treatment of dental caries, which involves the application of a 40% (w/v) aqueous SF solution to carious lesions, followed by dentine sealing using a glass ionomer cement preparation, is generally regarded to be safe. Nevertheless, fluoride toxicity represents one major clinical concern for its paediatric applications (62).

### 4.3 Potential involvement of silver nanoparticles in the therapeutic caries-arresting properties of SDF, SF and other silver(I) complexes

The MoAs of the beneficial clinical effects of SF and SDF complexes may also be at least partially ascribable to the actions of silver nanoparticles generated from these agents, or other silver(I) complexes, *in vivo*. Indeed, Ag(I) ions have a marked propensity for the generation of such mixed oxidation state Ag(0)/Ag(I)-containing silver nanoparticles, notably Ag(0)/AgCl-based species enshrouded by a wide range of salivary biomolecules, as found following treatment of WMSS samples with SDF and other Ag(I) complexes *in vitro* (14). Quite recently, Bertoglio et al. (63) explored the anti-bacterial and anti-biofilm actions of a silver nanoparticle preparation derived from a SF precursor, and using pectin as the reducing agent and protecting factor. These silver nanoparticles showed an enhanced bactericidal activity against *E. coli* PHL628 and *Staphylococcus epidermidis RP62A* (*S. epidermidis* RP62A), both as planktonic strains and as their corresponding biofilms. Fluoride ion enhanced the bactericidal effectiveness of solution/media-containing silver nanoparticles, even at levels as low as 10 µmol./L, a concentration at which it is completely inactive when applied alone.

One particularly potentially favourable feature of CSNPs resulting from the interactions of SDF with WMSSs is that a reducing agent for their generation is not required, i.e., they are autoconstructed in a time-dependent and light-exposure manner from the reaction of this agent with salivary electron-donors. Salivary reducing agents available for the conversion of Ag(I) to metallic Ag [Ag(0)] encompass L-cysteine [total mean salivary thiol level approximately 40 µmol./L in healthy individuals (64), both low-molecular-mass and protein-incorporated]; thiocyanate anion (SCN^−^), which is present at quite high mmol/L levels in human saliva, although it is higher in tobacco smokers since it arises from the metabolism of cigarette smoke hydrogen cyanide (65); the phenolic amino acid L-tyrosine (approximately 10 µmol./L) (66); and potentially any residual dietary reductants such as ergothioneine, ascorbate and further phenolics [very limited concentrations only, however, in view of the lengthy oral abstention episode required in Ref (14). for participant whole mouth saliva sample provision]. A similar “spectrum” of biomolecular electron-donors are also available in alternative oral fluids such as gingival crevicular fluid (GCF).

Until recently, the molecular interactions of SDF with carious dentin remained unexplored in view of difficulties associated with probing the nature of chemical reactions involved, since some of the most important ones involved solid phases. However, in 2017, a communication by Seto et al (67). reported the development of silver microwires of length 25–500 μm, and of 0.25–7.0 μm diameter, which were casted *in situ* within dentinal tubules following treatment of carious dental lesions with SDF solution *in vitro*. It was therefore postulated that such microwires, which displayed a diverse morphology down to a depth of 700 µm from the tooth surface, may serve as an Ag(0)-sourced Ag(I) “pool” for its microbicidal actions, the blockage of tubular flow, and an overall enhancement of lesion hardness. In addition to remineralisation processes, these data may also serve to provide valuable mechanistic clues regarding its clinical effectiveness. However, it remains a strong possibility that treatment with silver(I) complexes other than SDF, or CSNPs, may also give rise to such microwire formation.

In 2015, dos Santos Jr. et al (68). tested the caries-arresting properties of a rather sophisticated, loosely termed “nano silver fluoride” (NSF) formulation in 60 school children residing in a poor Brazilian community, of mean age 6.31 years. They found that application of NSF solution was more effective in hardening and arresting dental caries in primary teeth than the water placebo treatment, and that this property was similar to that observed with application of SDF once per annum. The NSF treatment application was simple and less expensive than SDF, and did not require a clinical setting. Moreover, this treatment was found not to have a “metallic” taste, and nor did it result in an unaesthetic, undesirable adverse dark/black discolouration of localised tissue.

This study involved a randomized controlled double-blind trial conducted on these children with active caries in primary teeth within a community location. The concentrations of each component in the silver-fluoride nanoparticle intervention material tested were chitosan 28,585 μg/ml; silver 376.5 μg/ml (equivalent to 3.49 mmol./L); and sodium fluoride 5,028.3 μg/ml (equivalent to nearly 120 mmol./L). Two drops of the nanoparticulate silver fluoride solution (33,989 mg/ml) were left in contact with tooth surfaces for 2 min. periods, this material being applied with a micro-brush (two drops were apparently equivalent to a 10 mg dose of the active ingredient). However, for the control group, only a single drop of water was applied; both treatments were performed only once during a 12-month duration. The presence of active caries as an outcome measure [using International Caries Detection and Assessment System (ICDAS II) criteria] was assessed at the one week, five- and 12-month trial duration time-points.

However, a critical evaluation of the chemistry of the therapeutic agent featured in this study revealed that the nanoparticulate Ag(I)-fluoride formulation applied was quite poorly defined, and little or no information on the product solution's stability and longevity throughout a 12-month trial period was provided. Indeed, the authors of the current study are fully aware that the composition and even morphology of such nanoparticulates are modified in a time-dependent manner [as described in Section 3.2 of Ref (14)], most especially when they are exposed to light: How was this controlled for and monitored in the reported study? This is of critical importance for a formulation applied for clinical use. The NSF material employed was actually a highly complex admixture featuring borohydride (BH_4_^−^)-reduced silver(I), with fluoride anion, along with a chitosan stabiliser, so this in itself is the source of many compositional complications. Does chitosan complex any residual Ag(I)? What else, besides Ag(I) ion, does the added BH_4_^−^ actually reduce? Therefore, this study formulation does not appear to have been sufficiently characterised for clinical use. Moreover, it has received some criticism, and confounding considerations relating to ethical and methodological approaches, along with those concerning safety of the formulation applied and its toxicological risks, have been reported for it (69). Since it does not appear to be a carefully-developed and -characterised treatment option, the positive results acquired in this study may not be directly comparable to the caries-arresting properties of SDF.

As might be expected, the Ag(I)/Ag(0) redox couple of Ag(I)-ligand/biomolecule complexes tightly regulates their anti-bacterial activities, i.e., the higher this value, the more easily the ligand facilitates the oxidation of Ag(0)-component nanoparticles to presumably more bioactive Ag(I) complexes. Previously, Navolotskaya et al. (70) investigated the effects of inorganic phosphate anion species on the oxidation of silver nanoparticles, which is clearly of much importance to oral health therapies which apply Ag(I)-containing agents such as SDF or SF in view of the availability of Ag(I)-complexing phosphate ligands in biosystems, and the potentially therapeutic generation of bactericidal Ag(I)-hydroxyapatite species *in vivo*. This process is also considered to be of much relevance to the production of CSNPs from the addition of SDF to human saliva (14), and potentially other oral fluids and biological sources too (71, 72). The experiments documented in Ref (70) discovered that in the presence of phosphate, the oxidation potential of the Ag(I)/Ag(0) couple is reduced to a less positive value than that observed in the absence of this agent, and that each class of phosphate anion exerted a differential effect on the rate and extent of Ag(0) oxidation, which was in the order PO_4_^3−^ >HPO_4_^2−^ >H_2_PO_4_^−^, i.e., it decreased with the decreasing electronic charge of these complexing anions (specifically an electrostatic influence). These results therefore clearly indicated a boosting of capacity for the oxidation of silver(0) with phosphate acting as a complexant for Ag(I). Since this oxidation process appears to be crucial for its bactericidal properties, then if sufficient concentrations of HPO_4_^2−^ are available at neutral salivary or acidotic carious dentin pH values within the oral environment, then they may facilitate this process, i.e., Ag(0) molecular oxidation dynamics in general, through its prior complexation of Ag(0)/Ag(I) species. These observations appear to be of relevance to our study reported in Ref (14), since the Ag/AgCl nanoparticulate deposits formed from the addition of SDF to human WMSSs contained detectable levels of available, potentially Ag(I)-complexing, phosphate ligands, along with the WMSS samples themselves (^31^P NMR analysis confirmed this). However, the thermodynamically-favourable conversion of Ag(I)-phosphate to AgCl (53, 54) negates against this. Nonetheless, Ag(I) ions can form a range of possible complexation species with inorganic phosphate anions, and these include AgPO_3_, Ag_3_PO_4_, Ag_2_HPO_4_, Ag_4_P_2_O_7_ and Ag_x_Na_(1−x)_PO_3_ (73). The silver(I) phosphate species Ag_3_PO_4_ has a very high stability constant of 8.89 × 10^17^ M^−1^ (74), and early information available on these Ag(I)-phosphate precipitation and speciation criteria are provided in Ref (75). The solubility of silver(I)-phosphate [as Ag(I)_3_PO_4_] in water is only 6.5 mg/L, equivalent to 15.5 µmol./L.

At the mean salivary pH value of 7.0 (29, 76), there is a slightly higher content of H_2_PO_4_^−^ over that of HPO_4_^2−^ present. However, at the typical acidotic pH value of carious dentin lesions (pH 4.5 (29), H_2_PO_4_^−^ is the predominant phosphate species present, with little or no HPO_4_^2−^ available. In principle, in the presence of phagocytically-generated hydrogen peroxide (H_2_O_2_), the aggressively reactive hydroxyl radical (^•^OH) may be generated from the oxidation or autoxidation of Ag(0) (Equations 5–7), and this action may itself be at least partially responsible for the antibacterial properties of colloidal silver preparations and formulations in view of the ability of ^•;^OH to cause a broad spectrum of damage to critical biomolecules such as DNA, along with localized cells and tissues (77).


(5) $$\mathrm{Ag}(0)\;+\;H_{2}O_{2}+®\mathrm{Ag}(I)\;{+}^{∙}\mathrm{OH}\;+\;OH^{-}$$

(6) $$\mathrm{Ag}(0)\;+\;O_{2}\to \;\mathrm{Ag}(I)\;+\;O_{2}^{∙-}$$

(7) $$O_{2}^{∙-}\;+\;2H^{+}\;+\;\mathrm{Ag}(0)\;\to \;H_{2}O_{2}\;+\;\mathrm{Ag}(I)$$

## 5 Conclusions and recommendations for future studies

In addition to unusual co-ordination geometries, the chemistry of silver, including its oxidation states, its complexation properties, and its nanoparticulate or solution status, is extremely diverse. Therefore, there remains much scope for exploitation regarding the applications of aqueous solutions of its coordination complexes or compounds as metallo-therapies, or as nanoparticulate biomedical materials, for oral health applications.

On the basis of this information, further investigations regarding the now increasingly extensive dental therapeutic applications of Ag(I) and Ag(I)-F complexes are required, with at least some emphasis placed on the circumvention or “masking” of the adverse dark brown-coloured silver(I) oxide (Ag_2_O) or black-coloured metallic silver [Ag(0)] formed (Figure 3); such unaesthetic dark-coloured depositional discolourations are frequently experienced during clinical application of these Ag(I) ion therapies. The deleterious foul-smelling ammoniacal odours commonly arising with the clinical employment of SDF also remain a serious concern, from both aesthetic and toxicological standpoints. Of notable consideration, bioactive, microbicidal silver(I)-complexes, along with CSNP materials, which may indeed be derived therefrom *in vivo*, should be further explored.

Of much interest, following the addition of SDP, SF or silver(I)-nitrate to WMSS samples, the autoconstruction of Ag/AgCl-based CSNPs via the IV-SCAN process, which also involves their interactions with encapsulating salivary biomolecules, may represent a valuable starting focal point for such future investigations. For these materials, no reducing agent is required for their generation, since they are biosynthesised in WMSS samples *in vitro* through the actions of one or more bioactive salivary reducing agents. This is a process which is also projected to occur in porous demineralised enamel, or dentin, these localisations providing sufficient time for any AgCl or other Ag(I) complexes to be converted to CSNPs via their reduction to Ag(0) by salivary electron-donors *in vivo*, although their synchronous photoreduction also remains an albeit limited possibility, despite the predominant absence of light within the oral environment. This mechanism serves as a viable alternative to the prior generation of insoluble Ag(I)-phosphate species from the reactions of Ag(I) with hydroxyapatite, followed by their reduction to dark-coloured Ag(0)-containing deposits (Figure 3).

To date, therapeutically-active nanoparticulate materials containing combinations of silver(0), silver(I) and fluoride have been commonly incorporated into dental restorative materials; however, optimisation of suitable delivery vehicles and, where relevant, the morphological characteristics and particle sizes of such preparations, require much further research investigations. Indeed, the employment of such agents for the treatment of deep carious cavities will also require a full scope of cellular toxicity and dose-linked safety evaluations in order to avoid any pulp irritation and further adverse events. Hence, full-scale studies of the biological chemistry, dispositional status and overall fates of silver(I), SF and SDF complexes in the oral environment should be optimized, along with those focused on the synthesis, characterization and further development of new and novel SF products for the arrest of dental caries. These, in turn, should provide much valuable biomolecular information regarding their therapeutic activities and MoAs (78).

## References

[1] Gao SS Amarquaye G Arrow P Bansal K Bedi R Campus G Global oral health policies and guidelines: using silver diamine fluoride for caries control. Front Oral Health. (2021) 2:685557. 10.3389/froh.2021.685557 35048029 PMC8757897

[2] Gao SS Zhao IS Hiraishi N Duangthip D Mei ML Lo ECM Clinical trials of silver diamine fluoride in arresting caries among children: a systematic review. JDR Clin Trans Res. (2016) 1(3):201–10. 10.1177/2380084416661474 30931743

[3] Ballikaya E Ünverdi GE Cehreli ZC. Management of initial carious lesions of hypomineralized molars (MIH) with silver diamine fluoride or silver-modified atraumatic restorative treatment (SMART): 1-year results of a prospective, randomized clinical trial. Clin Oral Investig. (2022) 26(2):2197–205. 10.1007/s00784-021-04236-5 34743243 PMC8572062

[4] Yamashita H. Penetration of ag(NH_3_)_2_F applied to an infected root canal. Gifu Shika Gakkai Zasshi. (1985) 12(3):417–39. (in Japanese). 3869606

[5] Liu Y Zhai W Du F. Clinical observation of treatment of tooth hypersensitiveness with silver ammonia fluoride and potassium nitrate solution. Zhonghua Kou Qiang Yi Xue Za Zhi. (1995) 30(6):352–4. (in Chinese). 8762541

[6] Rosenblatt A Stamford TC Niederman R. Silver diamine fluoride: a caries “silver-fluoride bullet”. J Dent Res. (2009) 88(2):116–25. 10.1177/0022034508329406 19278981

[7] World Health Organization Model List of Essential Medicines—22nd List, 2021. Geneva: World Health Organization (2021). (WHO/MHP/HPS/EML/2021.02). Licence: CC BY-NC-SA 3.0 IGO.

[8] Crystal YO Janal MN Yim S Nelson T. Teaching and utilization of silver diamine fluoride and Hall-style crowns in US pediatric dentistry residency programs. J Am Dent Assoc. (2020) 151(10):755–63. 10.1016/j.adaj.2020.06.022 32979954 PMC7510543

[9] Jiang CM Duangthip D Chan AKY Tamrakar M Lo ECM Chu CH. Global research interest regarding silver diamine fluoride in dentistry: a bibliometric analysis. J Dent. (2021) 113:103778. 10.1016/j.jdent.2021.103778 34391874

[10] Green E. A clinical evaluation of two methods of caries prevention in newly-erupted first permanent molars. Australian Dental J. (1989) 34:407–9. 10.1111/j.1834-7819.1989.tb00696.x 2818298

[11] Gotjamanos T. Pulp response in primary teeth with deep residual caries treated with silver fluoride and glass ionomer cement (‘atraumatic’ technique). Aust Dent J. (1996) 41(5):328–34. 10.1111/j.1834-7819.1996.tb03142.x 8961607

[12] Turton B Horn R Durward C. Caries arrest and lesion appearance using two different silver fluoride therapies on primary teeth with and without potassium iodide: 12-month results. Clin Exp Dent Res. (2021) 7(4):609–19. 10.1002/cre2.367 33370847 PMC9632638

[13] Peng JJ-Y Botelho MG Matinlinna JP. Silver compounds used in dentistry for caries management: a review. J Dentistry. (2012) 40(7):531–41.ISSN 0300-5712, 10.1016/j.jdent.2012.03.009 22484380

[14] Hunwin K Page G Edgar M Botana A Armitage R Bhogadia M Explorations of the chemical constitution and aqueous solution status of caries-arresting silver(I)-diammine fluoride and silver(I)-fluoride products using high-resolution ^19^F NMR analysis. Spectroscopic and SEM investigations of their interactions with human saliva: evidence for the *in vivo* salivary-catalysed autoconstruction of ag/AgCl-based nanoparticles (IV-SCAN)—part I. Front Oral Health. (2024) 5:1373885. 10.3389/froh.2024.1373885 38933119 PMC11199528

[15] Holloway CE Melnik M Nevin WA Liu W. Silver coordination and organometallic compounds: classification and analysis of crystallographic and structural data. J Coordin Chem. (1995) 35(1-2):85–178. 10.1080/00958979508033088

[16] Patai S. Chemistry of Organic Derivatives of Gold and Silver. New York: John Wiley & Sons (1999).

[17] Schmidbaur H. The fascinating implications of new results in gold chemistry. Gold Bull. (1990) 23:11–21. 10.1007/BF03214710

[18] Grootveld MC Razi MT Sadler PJ. Progress in the characterization of gold drugs. Clin Rheumatol. (1984) 3(S1):5–16. 10.1007/BF03342617 6432413

[19] Healy PC Mills NK White AH. Lewis-base adducts of group 1B metal (I) compounds. Part 11. Synthesis and crystal structure of adducts of silver (I) bromide with monomethyl-substituted pyridine bases. J Chem Soc Dalton Trans. (1985) (1):111–6. 10.1039/dt9850000111

[20] Housecroft CE. Silver 1992. Coord Chem Rev. (1996) 152(8):87. 10.1016/0010-8545(96)01269-6

[21] Schmidbaur H. Gold Progress in Chemistry, Biochemistry, and Technology. West Sussex, England: Wiley (1999).

[22] Puddephatt R. The Chemistry of Gold. Amsterdam, the Netherlands: Elsevier (1978).

[23] Fox BS Beyer MK Bondybey VE. Coordination **c**hemistry of **s**ilver **c**ations. J Am Chem Soc. (2002) 124(45):13613–23. 10.1021/ja0176604 12418916

[24] Bjerrum J. Metal amine formation in solution. XXVI. Stability constant and UV absorption spectrum of the triammine silver (I) complex. Acta Chemica Scandinavica, Series A. (1986) 40:392. 10.3891/acta.chem.scand.40a-0392

[25] Tyrra W. Silver(I) fluoride and related compounds in chemical synthesis. Heteroatom Chem. (2002) 13:561–6. 10.1002/hc.10102

[26] Cametti M Rissanen K. Recognition and sensing of fluoride anion. Chem Commun. (2009):2809–29. 10.1039/b902069a 19436879

[27] Zhan C-G Dixon DA. Hydration of the fluoride anion: structures and absolute hydration free energy from first-principles electronic structure calculations. J Phys Chem A. (2004) 108(11):2020–9. 10.1021/jp0311512

[28] Deane PA. The fluoride complexity of sc(III), cu(II), pb(II), zn(II), hg(II), Hg2(II), sn(II) and ag(I) in aqueous solution). Lawrence Berkeley National Laboratory (1955). Available online at: https://escholarship.org/uc/item/2fq44957 (Publication Date 1955-04-01).

[29] Silwood CJL Lynch EJ Seddon S Sheerin A Claxson AWD Grootveld MC. ^1^H NMR analysis of microbial-derived organic acids in primary root carious lesions and saliva. NMR Biomed. (1999) 12:345–56. 10.1002/(SICI)1099-1492(199910)12:6<345::AID-NBM580>3.0.CO;2-C 10516616

[30] Palmer WG. Experimental Inorganic Chemistry. United Kingdom: Cambridge University Press (1954). ISBN 9780521059022.

[31] Newton JJ Brooke AJ Duhamel B Pulfer JM Britton R Friesen CM. Fluorodesulfurization of thionobenzodioxoles with silver(I) fluoride. J. Organic Chem. (2020) 85(20):13298–305. 10.1021/acs.joc.0c01729 32924485

[32] Clements RM Barrow RF. The absorption spectrum of gaseous silver fluoride. Chem Commun. (London). (1968) 1:27–8. 10.1039/c19680000027

[33] Lozinšek M Vivoda MB Dragomir M. Crystal structure reinvestigation of silver(I) fluoride, AgF. Inorganic Comp. (2023) 8(1):x230018. ISSN 2414-3146. 10.1107/S2414314623000184 PMC9912324 36794053

[34] Craig JDC Brooker MH. On the nature of fluoride ion hydration. J. Solution Chem. (2000) 29:879–88. 10.1023/A:1005130616350

[35] Mei L Chu CH Lo ECM Samaranayake LP. Fluoride and silver concentrations of silver diammine fluoride solutions for dental use. Int J Paed Dent. (2013) 23:279–85. 10.1111/ipd.12005 23033939

[36] Gotjamanos T Afonso F. Unacceptably high levels of fluoride in commercial preparations of silver fluoride. Austral Dent J. (1997) 42:52–3. Available online at: https://doi-org.proxy.library.dmu.ac.uk/10.1111/j.1834-7819.1997.tb00097.x 10.1111/j.1834-7819.1997.tb00097.x 9078648

[37] Lindqvist I. On the crystal structure of silver thiocyanate. Acta Crystallogr. (1957) 10(1):29–32. 10.1107/S0365110X57000067

[38] Alharbi DA Almugren WM. An overview of silver diamine fluoride in pediatric dentistry. Saudi J Oral Dent Res. (2020) 5(1):5–10. 10.36348/sjodr.2020.v05i01.002

[39] Yamaga R Yokomizo I. Arrestment of caries of deciduous teeth with diamine silver fluoride. Dent Outlook Archieve. (1969) 33:1007–13.

[40] Yamaga R Nishino M Yoshida S Yokomizo I. Diammine silver fluoride and its clinical application. J Osaka Univ Dent Sch. (1972) 12:1–20. 4514730

[41] Suzuki T Nishida M Sobue S Moriwaki Y. Effects of diamine silver fluoride on tooth enamel. J Osaka Univ Dent Sch. (1974) 14:61–72. 4535096

[42] Lou YL Botelho MG Darvell BW. Reaction of silver diamine fluoride with hydroxyapatite and protein. J Dent. (2011) 39(9):612–8. ISSN 0300-5712. 10.1016/j.jdent.2011.06.008 21745530

[43] Peng J-Y Tsoi JK-H Matinlinna JP Botelho MG. Silver deposition on demineralized dentine surface dosed by silver diammine fluoride with different saliva. J Invest Clin Dent. (2019) 10:e12382. 10.1111/jicd.12382 30556962

[44] Turton B. Arrest of caries using different silver fluoride solutions in the healthy kids Cambodia project. ISRCTN. (2019). ISRCTN87596444. 10.1186/ISRCTN87596444 (last edited 21/02/2019). accessed 21/03/2020.

[45] Chu C-H Lo E. Microhardness of dentine in primary teeth after topical fluoride applications. J. Dent. (2008) 36:387–91. 10.1016/j.jdent.2008.02.013 18378377

[46] Mei ML Ito L Cao Y Lo EC Li QL Chu CH. An ex vivo study of arrested primary teeth caries with silver diamine fluoride therapy. J Dent. (2014) 42(4):395–402. 10.1016/j.jdent.2013.12.007 24373856

[47] Buzalaf MAR Pessan JP Honório HM Ten Cate JM. Mechanisms of action of fluoride for caries control. Monogr Oral Sci. (2011) 22:97–114. 10.1159/000325151 21701194

[48] Wu L Yang F. Comparison of the effects of three fluoride-containing agents on the demineralization of deciduous teeth *in vitro*. J Mod Stomatol. (2002) 16:216–8.

[49] Rosas SGP Téllez MÁA Espinoza EV. *In vitro* efficiency of fluoride-containing compounds on remineralization of carious enamel lesions under cyclic pH conditions. Rev Odontol Mex. (2014) 18(2):96–104. 10.1016/S1870-199X(14)72058-0

[50] Featherstone JD. Prevention and reversal of dental caries: role of low level fluoride. Community Dent Oral Epidemiol. (1999) 27(1):31–40. 10.1111/j.1600-0528.1999.tb01989.x 10086924

[51] Yu DG Kimura Y Fujita A Hossain M Kinoshita JI Suzuki N Study on acid resistance of human dental enamel and dentin irradiated by semiconductor laser with ag(NH_3_)_2_F solution. J Clin Laser Med Surg. (2001) 19(3):141–6. 10.1089/10445470152927973 11469306

[52] Mei ML Ito L Cao Y Li QL Lo EC Chu CH. Inhibitory effect of silver diamine fluoride on dentine demineralisation and collagen degradation. J Dent. (2013) 41(9):809–17. 10.1016/j.jdent.2013.06.009 23810851

[53] Mei ML Nudelman F Marzec B Walker JM Lo ECM Walls AW Formation of fluorohydroxyapatite with silver diamine fluoride. J Dent Res. (2017) 96(10):1122–8. 10.1177/0022034517709738 28521107 PMC5582683

[54] Mei ML Lo ECM Chu CH. Arresting dentine caries with silver diamine fluoride: what’s behind it? J Dent Res. (2018) 97(7):751–8. 10.1177/0022034518774783 29768975

[55] Da Costa-Silva CM Ambrosano GM Jeremias F De Souza JF Mialhe FL. Increase in severity of molar-incisor hypomineralization and its relationship with the colour of enamel opacity: a prospective cohort study. Int J Paediatr Dent. (2011) 21(5):333–41. 10.1111/j.1365-263X.2011.01128.x 21470321

[56] Zheng FM Yan IG Duangthip D Gao SS Lo ECM Chu CH. Silver diamine fluoride therapy for dental care. Jpn Dent Sci Rev. (2022) 58:249–57. 10.1016/j.jdsr.2022.08.001 36097560 PMC9463534

[57] Lansdown AB. Silver. I: its antibacterial properties and mechanism of action. J Wound Care. (2002) 11:125–30. 10.12968/jowc.2002.11.4.26389 11998592

[58] Sayed M Hiraishi N Matin K Abdou A Burrow MF Tagami J. Effect of silver-containing agents on the ultra-structural morphology of dentinal collagen. Dent Mater. (2020) 36(7):936–44. ISSN 0109-5641. 10.1016/j.dental.2020.04.028 32475750

[59] Maity K Panda DK Lochner E Saha S. Fluoride-induced reduction of ag(I) cation leading to formation of silver mirrors and luminescent ag-nanoparticles. J Am Chem Soc. (2015) 137(8):2812–5. 10.1021/ja512020w 25669417

[60] King EL Krall HJ Pandow ML. Studies pertaining to soluble silver iodide species. J Am Chem Soc. (1952) 74(14):3492–6. 10.1021/ja01134a012

[61] Gao SS Duangthip D Wong MCM Lo ECM Chu CH. Randomized trial of silver nitrate with sodium fluoride for caries arrest. JDR Clin Translat Res. (2019) 4(2):126–34. 10.1177/2380084418818482 30931709

[62] Duffin S Duffin M Grootveld M. Revisiting fluoride in the twenty-first century: safety and efficacy considerations. Front Oral Health—Preventive Dentistry. (2022) 3. 10.3389/froh.2022.87315 PMC9289262 35860375

[63] Bertoglio F De Vita L D'Agostino A Diaz Fernandez Y Falqui A Casu A Increased antibacterial and antibiofilm properties of silver nanoparticles using silver fluoride as precursor. Molecules. (2020) 25(15):3494. 10.3390/molecules25153494 32751978 PMC7436145

[64] Karim S Pratibha PK Kamath S Bhat GS Kamath U Dutta B Superoxide dismutase enzyme and thiol antioxidants in gingival crevicular fluid and saliva. Dent Res J (Isfahan). (2012) 9(3):266–72. 23087730 PMC3469891

[65] Tsuge K Kataoka M Seto Y. Cyanide and thiocyanate levels in blood and saliva of healthy adult volunteers. J Health Sci. (2020) 46(5):343–50. 10.1248/jhs.46.343

[66] Nakamura Y Kodama H Satoh T Adachi K Watanabe S Yokote Y Diurnal changes in salivary amino acid concentrations. In Vivo. (2010) 24(6):837–42. 21164041

[67] Seto J Horst JA Parkinson DY Frachella JC DeRisi JL. Silver microwires from treating tooth decay with silver diamine fluoride. bioRxiv. (2017):152199. 10.1101/152199

[68] dos Santos VE Jr Vasconcelos Filho A Targino AGR Flores MAP Galembeck A Caldas AF Jr A new “silver-bullet” to treat caries in children–nano silver fluoride: a randomised clinical trial. J. Dent. (2014) 42:945–51. 10.1016/j.jdent.2014.05.017 24930870

[69] Burns J Hollands K. Nano silver fluoride for preventing caries. Evid. Based Dent. (2015) 16:8–9. 10.1038/sj.ebd.6401073 25909929

[70] Navolotskaya DV Toh HS Batchelor–McAuley C Compton RG. Voltammetric study of the influence of various phosphate anions on silver nanoparticle oxidation. Chemistry Open. (2015) 4:595–9. 10.1002/open.201500100 26491638 PMC4608526

[71] Guo X Mahmud S Zhang X Yu N Faridul Hasan KM. One-pot green synthesis of ag@AgCl nanoparticles with excellent photocatalytic performance. Surface Innovations. (2021) 9(5):277–84. 10.1680/jsuin.20.00089

[72] Spagnoletti FN Kronberg F Spedalieri C Munarriz E Giacometti R. Protein corona on biogenic silver nanoparticles provides higher stability and protects cells from toxicity in comparison to chemical nanoparticles. J Environ Management. (2021) 297:113434. ISSN 0301-4797. 10.1016/j.jenvman.2021.113434 34400389

[73] Weast RC. CRC Handbook of Chemistry and Physics 1974–1975. 55th ed. Ohio, USA: CRC Press (1978). B135.

[74] Dean A. Lange’s Handbook of Chemistry. 15th ed. New York, NY, USA: McGraw-Hill, Inc. (1999).

[75] Firsching FH. Precipitation of silver phosphate from homogenous **s**olution. Anal. Chem. (1961) 33(7):873–4. 10.1021/ac60175a018

[76] Grootveld M Page G Bhogadia M Edgar M. Updates and original case studies focused on the NMR-linked metabolomics analysis of human oral fluids part I: emerging platforms and perspectives. Appl Sci. (2022) 12(3):1235. 10.3390/app12031235

[77] Halliwell B Grootveld M. The measurement of free radical reactions in humans. FEBS Lett. (1987) 231(1):9–14. 10.1016/0014-5793(87)81455-2 3030811

[78] Lansdown AB. Silver in health care: antimicrobial effects and safety in use. Curr Probl Dermatol. (2006) 33:17–34. 10.1159/000093928 16766878

